# Efficient Photoelectrochemical Water Splitting by Tailoring MoS_2_/CoTe Heterojunction in a Photoelectrochemical Cell

**DOI:** 10.3390/nano10122341

**Published:** 2020-11-26

**Authors:** Effat Sitara, Habib Nasir, Asad Mumtaz, Muhammad Fahad Ehsan, Manzar Sohail, Sadia Iram, Syeda Aqsa Batool Bukhari

**Affiliations:** School of Natural Sciences, National University of Sciences and Technology, H-12, Islamabad 44000, Pakistan; effat.sitara@sns.nust.edu.pk (E.S.); asad.mumtaz@sns.nust.edu.pk (A.M.); m.fahad.ehsan@sns.nust.edu.pk (M.F.E.); manzar.sohail@sns.nust.edu.pk (M.S.); sadia.iram@sns.nust.edu.pk (S.I.); aqsa.batool@sns.nust.edu.pk (S.A.B.B.)

**Keywords:** photoelectrochemical water splitting, heterojunction, molybdenum disulfide, cobalt telluride

## Abstract

Solar energy conversion through photoelectrochemical water splitting (PEC) is an upcoming promising technique. MoS_2_/CoTe heterostructures were successfully prepared and utilized for PEC studies. MoS_2_ and CoTe were prepared by a hydrothermal method which were then ultrasonicated with wt. % ratios of 1:3, 1:1 and 3:1 to prepare MoS_2_/CoTe (1:3), MoS_2_/CoTe (1:1) and MoS_2_/CoTe (3:1) heterostructure, respectively. The pure materials and heterostructures were characterized by XRD, UV–vis-DRS, SEM, XPS, PL and Raman spectroscopy. Photoelectrochemical measurements were carried out by linear sweep voltammetry and electrochemical impedance spectroscopic measurements. A maximum photocurrent density of 2.791 mA/cm^2^ was observed for the MoS_2_/CoTe (1:1) heterojunction which is about 11 times higher than the pristine MoS_2_. This current density was obtained at an applied bias of 0.62 V vs. Ag/AgCl (1.23 V vs. RHE) under the light intensity of 100 mW/cm^2^ of AM 1.5G illumination. The enhanced photocurrent density may be attributed to the efficient electron–hole pair separation. The solar to hydrogen conversion efficiency was found to be 0.84% for 1:1 MoS_2_/CoTe, signifying the efficient formation of the p-n junction. This study offers a novel heterojunction photocatalyst, for PEC water splitting.

## 1. Introduction

The sun is a huge source of energy. It is important to explore new ways of utilizing solar energy so that the problem of energy crises and global warming through environmental emission of gasses can be addressed [1]. Using solar energy to convert water into oxygen and hydrogen is a promising approach to remove energy shortages and environmental pollution [2]. Water splitting through a photoelectrochemical method has drawn tremendous attention because it is pollution and carbon-free technology [3,4]. The goal of clean energy can be achieved by the formation of heterostructures, thereby engineering the surface of the semiconductors [5,6]. One way to produce an effective photocatalyst is to use the p-n junction as a photoanode in the photoelectrochemical devices [7,8]. This p-n junction provides a medium or space-charge region where electrons from the n-type semiconductor and holes from the p-type semiconductor are mobilized [9]. This region launches an electrostatic field that helps to reduce the electron–hole recombination and enables easy transport of electrons [10].

Transition metal di-chalcogenides like molybdenum disulfide are being extensively studied now a days due to their excellent electrical [11,12,13], mechanical [14,15], photoelectrochemical [16,17], photocatalytic [18,19,20], optical [21,22], sensing [23,24,25] and photovoltaic [26,27,28] properties. MoS_2_ is an n-type semiconductor having a direct bandgap of 1.9 eV. Few layer MoS_2_ is considered to be a good photoanode for photoelectrochemical water splitting (PEC) due to its band gap in the solar absorption spectrum [29]. Its highly active edge sites display efficient photocatalytic activity [30]. To overcome the problem of recombination of electrons and holes, effective charge separation and charge transfer, various heterojunctions of MoS_2_ with different materials are reported till now, including the latest as ZnO/MoS_2_ [16], p-GaN/MoS_2_ [31], CdS@CoMo_2_S_4_/MoS_2_ [32], MnS/MoS_2_ [33] for PEC water splitting. The thrust for a better material continues and new materials are being reported every hour. However, its composite with cobalt telluride is not yet reported, to the best of our knowledge.

Cobalt telluride is a p-type semiconductor having a band gap of 2.15 eV [34]. It has shown good photocatalytic activity for CO_2_ reduction [35]. The composites of CoTe with Si-Microwires has also been used for solar water splitting [36]. Although, MoS_2_ and CoTe both have comparable band gaps with their specific intrinsic properties but their unique combination to develop an effective p-n junction is not reported yet. This study aims to understand few important factors in this novel MoS_2_/CoTe heterostructure which are, charge separation, electron–hole recombination, charge transfer and photoconversion efficiency for PEC water splitting. This novel heterojunction could be promising and is required to be explored in detail, under visible light irradiation for improved photoelectrochemical water splitting.

In this work, MoS_2_/CoTe p-n heterojunction was fabricated by a simple sonochemical method. The as-prepared heterostructures showed a high photoelectrochemical response towards water splitting. The heterostructures showed much better activity than the pure counterparts, due to the successful creation of the p-n junction and effective separation of the conduction and valence bands, thereby reducing the electron–hole recombination.

## 2. Results and Discussion

### 2.1. Phase Analysis

**Figure 1:**
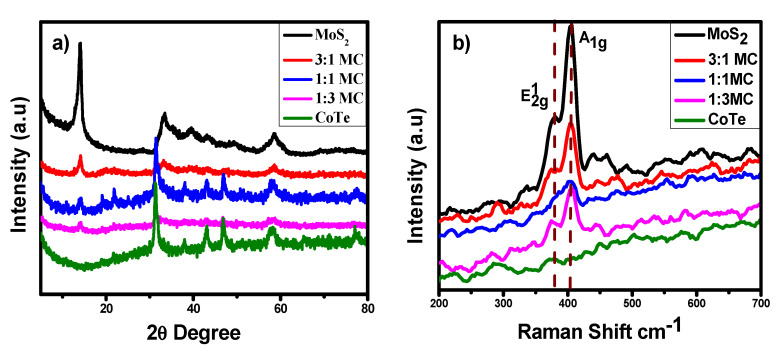
(**a**) X-ray diffraction pattern of CoTe, MoS_2_, 1:3 MoS_2_/CoTe (MC), 1:1 MC, 3:1 MC. (**b**) Raman shift values of MoS_2_, CoTe and their composites.

X-ray diffraction studies were carried out to approve the crystallinity and structures of the prepared photocatalysts as shown in Figure 1a. CoTe showed diffraction peaks at 31.4°, 43°, 47.2°, 57.5°, 58.5° and 56.7° corresponding to the (101), (102), (110), (201), (112) and (201) hexagonal phases and match with the standard JCPDS card No. 00-034-0420 [35]. On the other hand, diffraction peaks at 14°, 28.7°, 34.4°, 42.6° and 57.5° correspond to the (002), (103), (100), (105) and (110) planes of the hexagonal wurtzite structure of MoS_2_ [37] and match with the JCPDS card No. 37-1492 [38]. The exact matching of the peaks of CoTe and MoS_2_ with the standards show that there is no impurity present in the sample. The composites of MoS_2_ and CoTe with ratios of 1:3 MoS_2_/CoTe (1:3 MC), 1:1 MoS_2_/CoTe (1:1 MC) and 3:1 MoS_2_/CoTe (3:1 MC) show good correspondence with the pure MoS_2_ and CoTe. The peak intensity of the characteristic peak of MoS_2_ at 14° decreases with increase in the ratio of CoTe, i.e., 3:1 MC > 1:1 MC > 1:3 MC in the heterostructures. The heterostructure 1:1 MC shows characteristic peaks from both MoS_2_ and CoTe. This can be attributed to the successful formation of heterostructures.

### 2.2. Raman Analysis

To further understand the microstructure and the crystallinity of the photocatalysts, Raman spectra were taken as shown in Figure 1b. In Raman spectra of MoS_2_, two peaks are very prominent at around 380 and 405 cm^−1^ which match the E^1^_2g_ and A_1g_ in-plane vibration of Mo and S atoms and out of plane vibrations of sulfur atoms from pure 2H-MoS_2_ [39], respectively. The difference between the peaks of E^1^_2g_ and A_1g_ is 24 cm^−1^ which is comparable to previously reported four-layered MoS_2_ [40]. However, CoTe did not show any peak. While the MoS_2_/CoTe with different ratios shows the effect of compositional modifications while forming a heterojunction. The intensity of E^1^_2g_ and A_1g_ peaks of MoS_2_ is being supported by increasing the composition of CoTe in the heterojunction. This illustrates the effective interfacial interaction between the individual components of the heterojunction required for the efficient charge transfer phenomenon during photocatalytic processes.

### 2.3. Structural and Compositional Analysis

**Figure 2:**
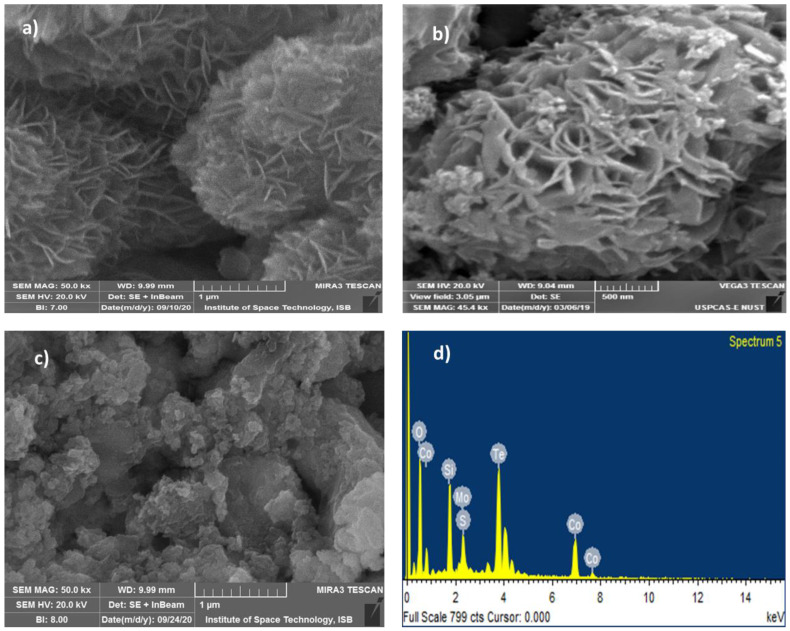
SEM images of (**a**) MoS_2_ at 1 µm, (**b**) 1:1 MC at 500 nm, (**c**) CoTe at 1 µm, (**d**) EDS spectra of 1:1 MC.

Morphology of the as-synthesized photocatalysts was studied through scanning electron microscopy (SEM) shown in Figure 2. The flower-like structure of MoS_2_ is observed in Figure 2a, well-matched with the previously reported flowerlike nanosheets of MoS_2_ [41]. While Figure 2b for 1:1 MC shows the successful decoration of CoTe nanostructures on the surface of the flower-like structure with highly porous features of MoS_2_. The morphology shows the homogeneous mixing of two components in the heterojunction of 1:1 MC. This homogeneity with its effective interfacial contact developed by using the sonochemical method is beneficial in photocatalysis during the charge transfer phenomenon from CoTe to MoS_2_. In Figure 2c, the pure CoTe showed a semicircular and agglomerated morphology mainly due to the magnetic properties of CoTe [36]. SEM images of 3:1 MC and 1:3 MC are shown in Figure S1.

Energy-dispersive X-ray spectroscopy (EDS) helps to find the purity and elemental composition of the prepared materials [42]. The EDS spectra in Figure 2d show the elemental composition of 1:1 MC. The peaks of Mo, S, Co and Te are clearly visible. The peaks for Si and O are due to the glass slides used for the sample preparation for SEM analysis. Figure S2a–d show there are no impurities found in the formation of pure materials and composites, which demonstrates the purity of the synthesized materials.

### 2.4. XPS Analysis

**Figure 3:**
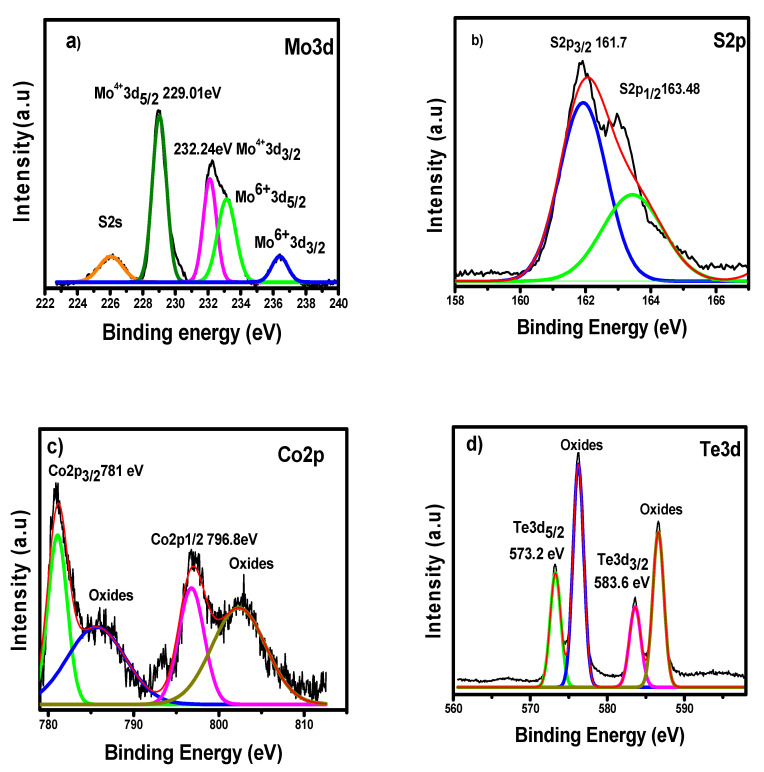
XPS spectra of MoS_2_ (**a**) Mo3d, (**b**) S2p and CoTe (**c**) Co2p, (**d**) Te3d.

Oxidation states and chemical composition of the prepared materials were established by employing the X-ray photoelectron spectroscopy. XPS spectra of Mo3d and S2p are shown in Figure 3a,b. The Mo^4+^ oxidation state was confirmed by the binding energies for Mo3d_5/2_ at 229.01 eV and Mo3d_3/2_ at 232.24 eV [43]. The presence of sulfur can be established by the peak at 161.7 eV for S2p_3/2_ orbital and 163.48 eV for S2p_1/2_ [17]. The peaks positioned at 233.1 eV and 236.2 eV can be ascribed to the Mo^+6^ 3d_5/2_ and Mo^+6^ 3d_3/2_ oxidation state which appears due to minor oxidation of the edges during MoS_2_ synthesis [44,45]. Figure 3c shows peaks at 781 eV and 796.8 eV for Co 2p_3/2_ and 2p_1/2_, respectively, which appear due to the existence of the Co^2+^ oxidation state [46]. The peaks appearing in Figure 3d at 573.2 eV and 583.6 eV correspond to the Te3d_5/2_ and Te3d_3/2_, respectively, which show the Co^2+^ oxidation state of tellurium in the CoTe [36]. The other peaks are credited to the oxides of cobalt and tellurium which might be due to the reaction of the surface of CoTe with oxygen in the atmosphere [35].

### 2.5. Alignment of Energy Levels

The main features for the understanding of the mechanism of a photoelectrochemical process are the position of the valence and conduction bands and band gap against the standard hydrogen electrode (SHE) and they help to align the energy levels [47].

**Figure 4:**
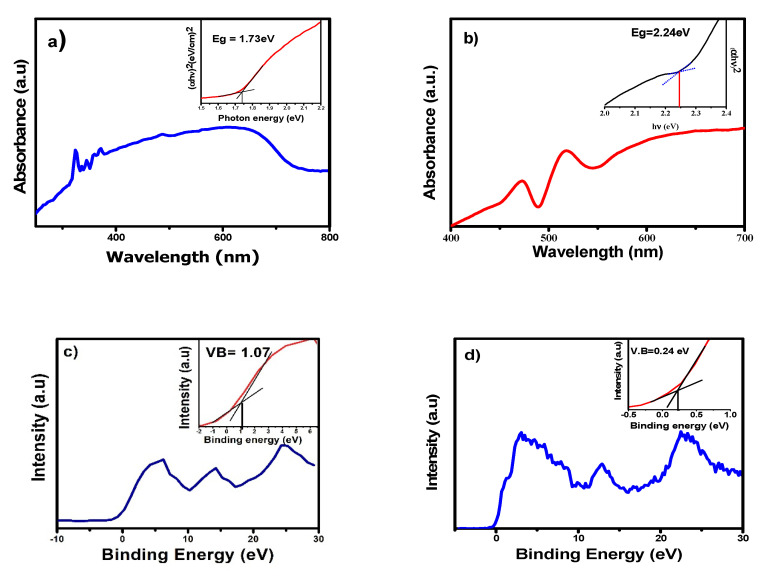
UV–vis-DRS spectrum of (**a**) MoS_2_, (**b**) CoTe with insets showing the respective Tauc plots and band gap of MoS_2_ and CoTe. (**c**) MoS_2_ and (**d**) CoTe are the XPS spectra for valence band positions in the corresponding insets.

UV-Vis diffused reflectance spectra(DRS) were obtained to evaluate the optical properties of the synthesized materials. Kubelka–Munk equation was employed to calculate the band gap by converting the diffused reflectance calculation to equivalent absorption coefficients of both MoS_2_ and CoTe. Tauc plots were plotted to calculate the band gap shown in Figure 4a. MoS_2_ came out with a band gap of 1.73 eV and CoTe with 2.24 eV as shown in Figure 4b.

**Figure 5:**
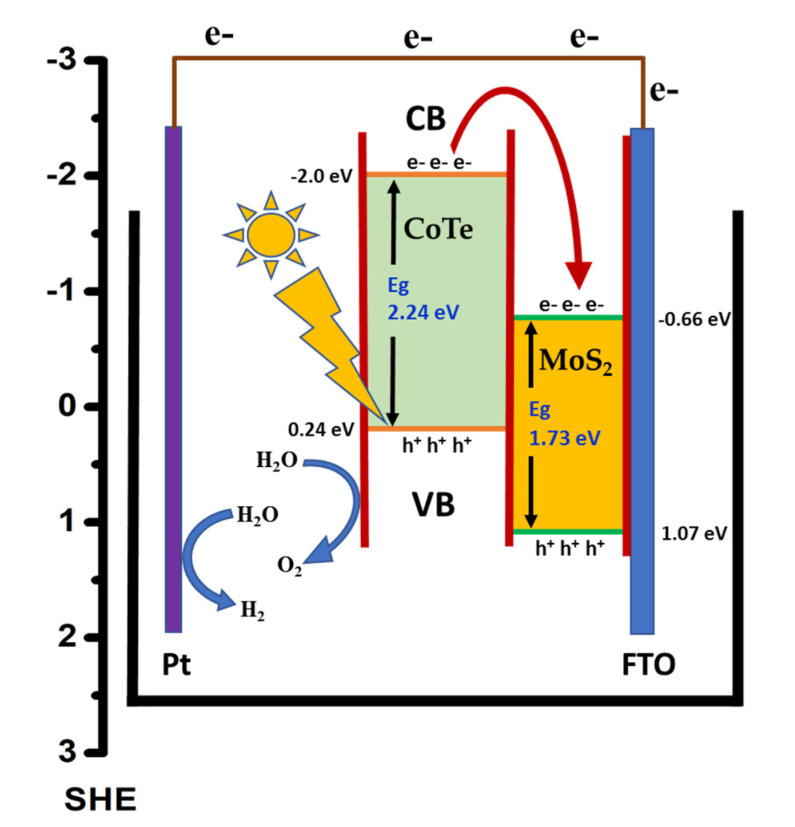
Schematic illustration of alignment of the energy level of MoS_2_/CoTe nanocomposite showing photoelectrochemical water splitting.

The XPS spectra for valence band show a valence band position for MoS_2_ in Figure 4c to be 1.07 eV and it shows the valence band position for CoTe as 0.24 eV in Figure 4d. The energy levels are aligned and drawn in accordance with these readings. The alignment of energy levels is shown in Figure 5.

### 2.6. Photoelectrochemical Analysis

**Figure 6:**
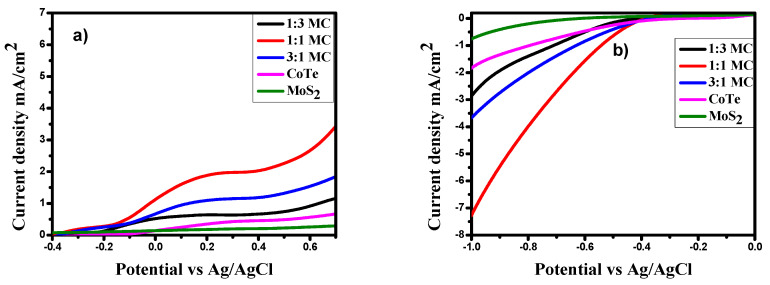

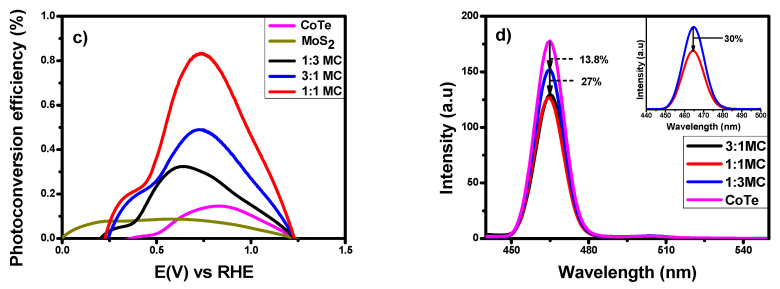
(**a**) I–V curves of pure MoS_2_, CoTe, 1:3 MC, 1:1 MC and 3:1 MC, for oxygen evolution. (**b**) I–V curves of pure MoS_2_, CoTe, 1:3 MC, 1:1 MC and 3:1 MC for hydrogen evolution. (**c**) Solar to hydrogen conversion efficiency of MoS_2_, CoTe, 1:3 MC, 1:1 MC and 3:1 MC. (**d**) PL spectrum of the pure CoTe, 3:1 MC 1:1 MC and 1:3 MC.

To examine the photoelectrochemical activity of the photocatalysts, linear sweep voltammograms were collected under illumination as shown in Figure 6. Pristine MoS_2_ and CoTe showed a photocurrent density of 0.26 mA/cm^2^ and 0.59 mA/cm^2^ at 0.62 V vs. Ag/AgCl (1.23 V vs. RHE) under visible light illumination of 100 mW/cm^2^ with AM 1.5 G, respectively. The photocurrent densities of MoS_2_/CoTe with 1:3 MC, 1:1 MC and 3:1 MC are found to be 0.97, 2.79 and 1.59 mA/cm^2^ at 1.23 V vs. RHE under visible light illumination intensity of 100 mW/cm^2^ are observed, respectively. Figure 6a shows the gradual increase in the photocurrent response in the form of a bump in peak for the 1:1 ratio of photocatalyst around the voltage of 0.24 V. This reveals that the photoexcited charges have moved from the valance band to the conduction band and a point of saturation is attained due to the maximum electron movement to the conduction band and fulfillment of the active sites on the surface of the photocatalyst. A maximum photocurrent density was observed in MoS_2_/CoTe with a 1:1 ratio which is 11 times higher than pristine MoS_2_ and four times greater than pristine CoTe. It is observed that Ohmic J-V curves originate in individual components of the heterojunction while MoS_2_/CoTe with a 1:1 ratio shows a standard peak of photoelectrochemical water splitting with its maximum saturation potential stabilization.

The onset potential is an important parameter while studying photoelectrochemical properties [48]. The observed onset potentials are −0.38 V, −0.3 V and −0.1 V vs. Ag/AgCl for 1:1 MC, MoS_2_ and CoTe, respectively, as shown in Figure 6a. The gradual slope increase can be attributed to jumping of the electrons from the valence band to the conduction band till the point of saturation, and then the sharp increase in photocurrent is observed. This shows efficient charge separation of electron–hole pair and transportation which results in the decrease of anodic onset potential [49]. The 1:1 MC shows an 80 mV decrease in onset potential in comparison to pristine MoS_2_. This decrease in onset potential rises to 280 mV in comparison to pristine CoTe. These results illustrate the synergistic effect of MoS_2_/CoTe heterojunction development with optimum 1:1 composition for maximum light harvesting. Moreover, the flower-like template of MoS_2_ provides a platform to CoTe for its efficient electron–hole pair separation and its transportation at the electrode–electrolyte interface in MoS_2_/CoTe heterojunction [50]. It was also evident that MoS_2_ contributes to the fast transportation of photoexcited charges as shown in the J-V characteristics curve of 1:1 MC and 3:1 MC in comparison to 1:3 MC. In another way, it is observed that pristine CoTe has poor kinetics in terms of charge transportation while, when it is mixed in proper composition in the heterojunction, it boosts the electron–hole pair charge separation and transport properties of the developed photocatalysts. A comparison of reported MoS_2_-based photoanodes with the current study is given in Table 1.

The cathodic current also shows a good response to the hydrogen evolution at the counter electrode as shown in Figure 6b. The pure CoTe and MoS_2_ exhibited a photocurrent density of −1.76 and −0.65 mA with an onset potential of −0.33 and −0.65 V vs. Ag/AgCl, respectively. The formation heterojunction changes the response of photocurrent for the composites. The 1:3 MC and 3:1 MC showed the maximum photocurrent density of −2.84 mA and −3.604 mA with an onset potential of −0.41 and −0.34 V vs. Ag/AgCl, respectively. The best results were observed with the 1:1 MC composite showing a photocurrent density of −7.21 mA with an onset potential of −0.36 V vs. Ag/AgCl. These outcomes also match with the results of anodic photocurrent response leading to a better formation of p-n heterojunction among CoTe and MoS_2_.

Solar to hydrogen conversion efficiency (STH) is presented as shown in Figure 6c. A lesser onset potential means less applied bias is required to gain optimum STH [55]. The STH is calculated by using Equation (1):

***η* = *I*(1.23 − *V*)/*P*in** (1)

where ***η*** is the STH, ***I*** is photocurrent density at the given potential, *V* is the applied potential vs. RHE, *P*in the intensity of the irradiating light at 100 mW/cm^2^ [6]. Pure MoS_2_ and CoTe exhibit a maximum conversion efficiency of 0.14 and 0.08% at an applied bias of 0.82 V and 0.36 V, respectively. Heterojunction MoS_2_/CoTe with 1:3 and 3:1 showed an STH efficiency of 0.325% and 0.48% at an applied bias of 0.65 V and 0.72 V vs. RHE, respectively. The highest STH efficiency was achieved with the 1:1 MC photocatalyst with 0.82% at an applied bias of 0.72 V vs. RHE. The composite heterojunction is working effectively with increased photocurrent response thereby increasing the STH efficiency. These results agree well with the J-V characteristics.

The electron–hole recombination and the efficiency of trapping the charge can be determined using the photoluminescence (PL) [56] as shown in Figure 6d. Figure 6d shows that pristine CoTe, 3:1 MC (13.8%) and 1:3 MC (27%) exhibited decrease in PL intensity as compared to 1:1 MC. It is evident that heterostructure 1:1 MC showed a 30% decrease as compared to the pure CoTe signal, so there is an effective charge transfer taking place through a successfully operating p-n junction. A facile electron transfer is taking place thus reducing the electron/hole recombination. These results are in agreement with the J-V characteristics of the photocatalysts and empowered the development of such type of heterojunctions to improve the photochemical properties of the component catalysts to integrate and deploy for efficient and smart solar cell devices and their large scale production.

**Figure 7:**
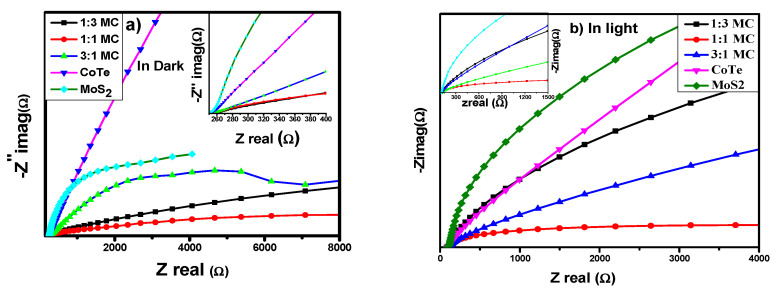

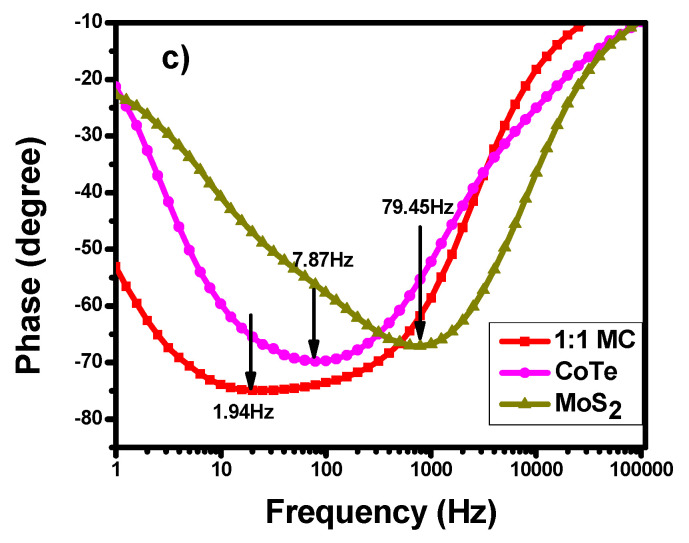
(**a**) Nyquist plot of the pure and composites in dark, (**b**) Nyquist plot of pure and composites in light and (**c**) Bode plot.

Electrochemical impedance spectroscopy was employed to have a profound understanding of the charge transfer mechanism in darkness (Figure 7a) and in light (Figure 7b) under open circuit potential (OCP) conditions. The curves show two semicircles in darkness in Figure 7a. Charge transfer resistance is associated with the first semicircle at the high-frequency region and mass transfer resistance is connected to the second at the low-frequency region [57]. Whereas the inset in Figure 7a displays the high-frequency region in dark, which signifies mass transfer to be the dominant phenomenon. Figure 7b displays the OCP conditions in light and there are no semicircles observed in the high-frequency region as shown in the inset of Figure 7b. This may be due to the charge transfer as a prominent process in presence of light [58]. Typically, the reduced diameter of the semicircle shows less amount of charge transfer resistance on the electrode and electrolyte interface [59]. Hence, the recombination decreases significantly in 1:1 MC heterojunction as suggested by LSV and PL results, showing effective charge transfer as compared to pristine components of heterojunction.

The Bode plot in Figure 7c shows the characteristic peak frequencies of the pure and 1:1 MC composite heterojunction. The characteristic peak frequency shifts to a lower value from MoS_2_ to CoTe to 1:1 MC, owing to the longer lifetime (1.94 Hz) of the photoexcited charges in heterojunction of 1:1 MC as compared to the pure counterparts [60]. These results are in agreement with J-V and PL studies and support the strategy of developing a potential p-n type of heterojunction with their comparable band gaps.

### 2.7. Mott–Schottky Plot

The Mott–Schottky plots of pristine MoS_2_, CoTe, 1:3 MC, 1:1 MC and 3:1 MC heterojunctions have been obtained and presented in Figure S3 (Supplementary Material). Mott–Schottky peaks of pure MoS_2_ and CoTe and the composites are shown in Figure S3a (Supplementary Material). The peaks show a trend of increasing n-character in the composites hence showing more PEC activity and formation of the p-n junction. The positive slope in Figure S3b (Supplementary Material) shows the n-type nature of MoS_2_. While the CoTe showed a negative slope in Figure S3c (Supplementary Matterial) owing to the p-type nature of CoTe semiconductor. This confirms the formation of an effective p-n junction viable for photoelectrochemical water splitting. Figure S4 shows a corresponding I–t curve at a potential of −0.4 V under the chopped light illumination. The photocurrent increases immediately from OFF to ON state proving that the present system is sensitive to light illumination and efficient in the generation and separation of electron–hole pairs through p-n junction to the counter electrode for water splitting.

## 3. Experimental

### 3.1. Synthesis of MoS_2_

MoS_2_ was synthesized using a hydrothermal method [61]. Sodium molybdate and thiourea (1:4 mole ratio) were dissolved in water (32 mL) and placed in Teflon lined stainless steel autoclave (40 mL). Then the autoclave was retained in an oven (220 °C) for 24 h. After the benchtop cooling, the obtained black powder was washed three times with deionized water and then with ethanol followed by vacuum drying (at 70 °C) for 24 h.

### 3.2. Synthesis of CoTe

Cobalt telluride was synthesized using a hydrothermal route [35]. During the synthesis procedure, tellurium powder and CoCl_2_ (1:1 mole ratio) were dissolved in 6 M KOH solution (32 mL). Hydrazine hydrate (8 mL) was added to this mixture and stirring was done for 1 h at room temperature. The mixture was transferred to a 40 mL Teflon lined autoclave. The autoclave was placed for 3 h (200 °C) in the oven. The precipitates were obtained and washed three times with deionized water and ethanol. The collected powder was dried in an oven (70 °C).

### 3.3. Development of MoS_2_/CoTe Heterojunction Using Sonochemical Method

**Scheme 1:**
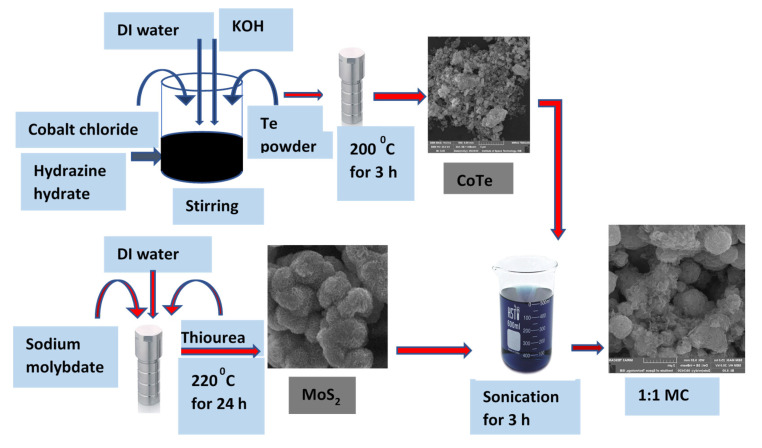
Schematic illustration of MoS_2_, CoTe and MoS_2_/CoTe heterostructure formation.

MoS_2_/CoTe heterojunctions were developed by the sonochemical method. The heterostructures were prepared with wt. % ratios of 1:3, 1:1 and 3:1 of MoS_2_ to CoTe. For a 1:1 ratio, MoS_2_ (50 mg) and CoTe (50 mg) were dispersed in ethanol (25 mL) under ultrasonic shaking for 3 h. Finally, the powder was vacuum dried (80 °C) for 10 h. Other ratios were made in the same manner. These heterostructures were used for photoelectrochemical water splitting. A schematic illustration of the formation mechanism is shown in Scheme 1.

### 3.4. Characterization

XRD analysis was recorded on an X-ray powder diffractometer [STOE Germany] by using Cu Kα at λ = 1.54 Å. UV–vis and DRS analyses were performed on Perkin Elmer [Massachusetts, USA] Lambda 365 spectrometer. VEGA3 TESCAN [Fuveau, France] scanning electron microscope in combination with an energy-dispersive X-ray spectroscope was used to obtain images and elemental composition of the photocatalysts at an acceleration voltage of 20 KV. Photoluminescence spectra were obtained by using Perkin Elmer [Massachusetts, USA] FL 6500/8500 spectrometer. The X-ray photoelectron spectroscopic analysis was carried out on the ESCALAB 250Xi [Massachusetts, USA] X-ray photoelectron spectrophotometer. Raman spectra were obtained using a BTC162E-532S-SYS [Newark, DE, USA] system with a 532 nm excitation wavelength.

### 3.5. Photoelectrochemical Measurements

Photoelectrochemical (PEC) studies were performed using a quartz cell made up of three electrode system. Platinum gauze was used as a counter electrode and Ag/AgCl as a reference electrode. FTO slides were used as a working electrode with a working area of 1 cm^2^. A 0.5 M sodium sulfate solution was used as an electrolyte to maintain the stability of the working sulfide electrode. A solar simulator [Solar Light Company, Inc. Glenside PA, USA] was used to carry out studies under illumination. A 300 W Xe lamp was used with a cut off filter of λ > 420 nm for the visible light source. The incident intensity of the light was calibrated and kept at 100 mW/cm^2^ of AM 1.5G using standard silicon solar cell (DyeSole). The photoelectrochemical measurements were taken on Gamry potentiostat [Philadelphia, PA, USA]. The photoelectrodes were prepared by making the sample ink. The ink was prepared by mixing 20 mg of the photocatalyst in 0.5 mL of DI water and adding 3 µL of Nafion as a binder. This mixture was ultrasonicated, to form a homogeneous ink, for 2 h. The as-prepared sample was drop casted on the FTO substrate (1 cm^2^ area) and air-dried for further studies.

## 4. Conclusions

In the present study, a novel and effective p-n heterojunction was designed between MoS_2_ and CoTe by a facile sonochemical method. MoS_2_ showed a very low photocurrent response of 0.26 mA, which on the formation of an effective p-n junction with CoTe, in a 1:1 ratio, raised to 2.79 mA. This is an increase of almost 11 times as compared to the pristine MoS_2_. The longer lifetime of the photoexcited charges and lessen recombination of electrons and holes was achieved and confirmed by the EIS and PL studies for 1:1 MC. Solar to hydrogen conversion efficiency also increased from 0.14% and 0.08% for MoS_2_ and CoTe, respectively to 0.84% for 1:1 MC. This novel heterostructure may be a potential candidate for efficient photoelectrochemical water splitting.

## Figures and Tables

**Table 1:** A comparison of reported MoS_2_-based photoanodes with the current study.

S.No.	Heterostructures	Method	Electrolyte pH	Photocurrent Density	Ref.
1.	MoS_2_/CoTe	Ultrasonication	0.5M Na_2_SO_4_ pH = 7	2.79 mA/cm^2^	This work
2.	ZnO@MoSx	Electrodeposition	0.5 M Na_2_SO_4_ pH = 7	1.42 mA/cm^2^	[51]
3.	NaNbO_3_/MoS_2_	Hydrothermal	0.5 M Na_2_SO_4_ pH = 7	3.56 mA cm^−2^	[52]
4.	In_2_Se_3_/MoS_2_	Ultrasonication	1 M Na_2_SO_4_ pH = 7	8 µA/cm^2^	[53]
5.	Au-MoS_2_	Stirring	0.1 M KH_2_PO_4_ pH = 7	790 μA/cm^2^	[29]
6.	MoS_2_/BiVO_4_	Hydrothermal	1 M Na_2_SO_4_ pH = 7	0.63 mA/cm^2^	[54]

## Supplementary Materials

The following are available online at https://www.mdpi.com/2079-4991/10/12/2341/s1, Figure S1 (Supplementary Material) shows the SEM spectra of 3:1 MC and 1:3 MC photocatalysts, Figure S2 shows EDS spectra of MoS_2_, CoTe, 3:1 MC and 1:3 MC. Figure S3 shows the Mott–Schottky plots for MoS_2_, CoTe, 3:1 MC, 1:1 MC and 1:3 MC. Figure S4 shows chronoamperometric response of light on/off for 1:1 MC.
