# Supplementary material for: Efficient Photoelectrochemical Water Splitting by Tailoring MoS2/CoTe Heterojunction in a Photoelectrochemical Cell
Source: Nanomaterials (Basel). 2020 Nov 26;10(12):2341. doi: 10.3390/nano10122341 (PMC7760392; doi:10.3390/nano10122341)
Supplement: Supplementary file 1 [file nanomaterials-10-02341-s001.pdf]

nanomaterials

Supplementary Materials

Efficient Photoelectrochemical Water Splitting by Tailoring MoS_2/CoTe Heterojunction in a Photoelectrochemical Cell

Effat Sitara, Habib Nasir *, Asad Mumtaz, Muhammad Fahad Ehsan, Manzar Sohail, Sadia Iram and Syeda Aqsa Batool Bukhari

School of Natural Sciences, National University of Sciences and Technology, H-12 Islamabad 44000, Pakistan; effat.sitara@sns.nust.edu.pk (E.S.); asad.mumtaz@sns.nust.edu.pk (A.M.); m.fahad.ehsan@sns.nust.edu.pk (M.F.E.); manzar.sohail@sns.nust.edu.pk (M.S.); sadia.iram@sns.nust.edu.pk (S.I.); aqsa.batool@sns.nust.edu.pk (S.A.B.B.)

* Correspondence: habibnasir@sns.nust.edu.pk

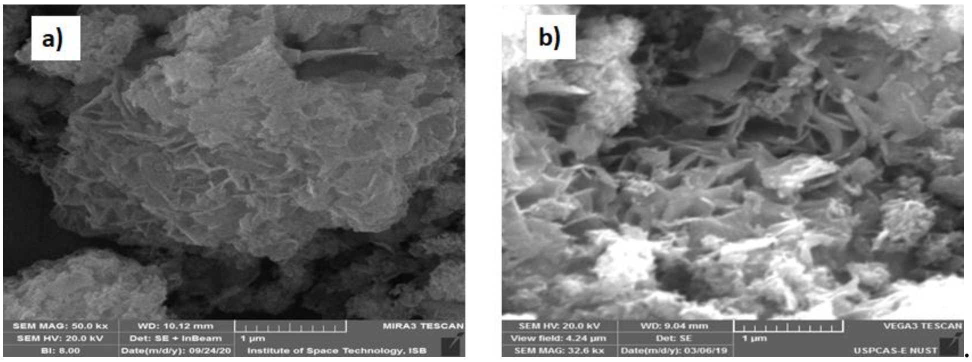

Figure S1. SEM images of (**a**) 3:1 MC and (**b**) 1:3 MC.

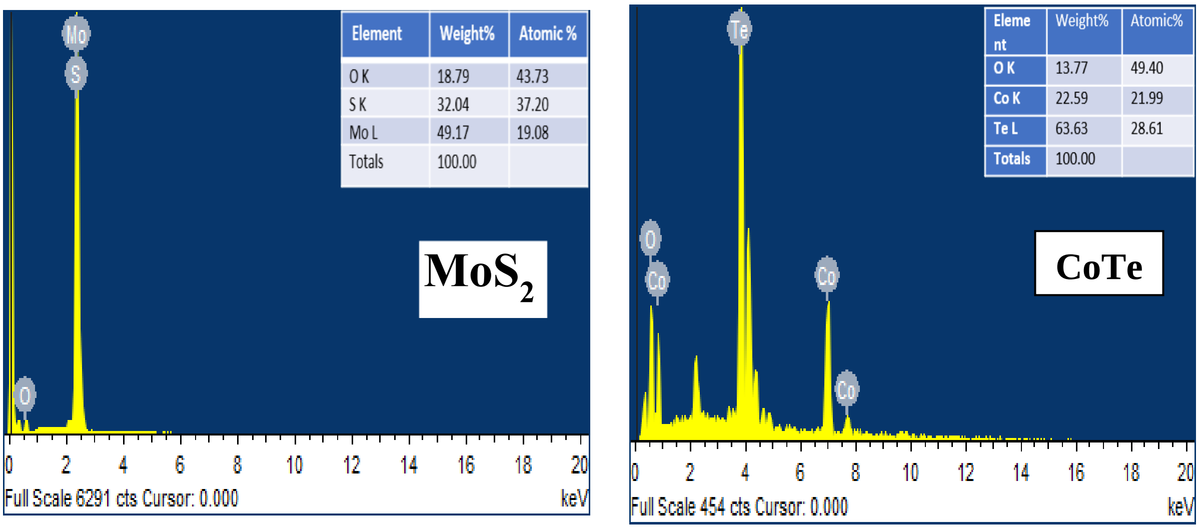

Figure S2. EDS spectra of MoS_2, CoTe, 3:1 MC and 1:3 MC.

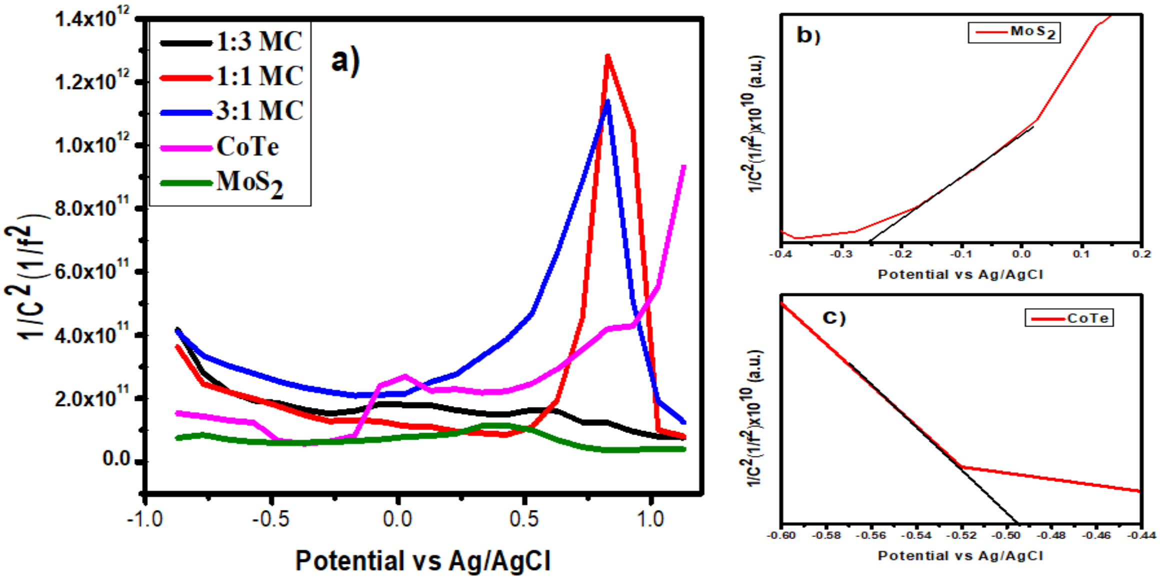

Figure S3. Mott-Schottky plot of (**a**) MoS_2, CoTe, 3:1 MC, 1:1 MC and 1:3 MC (**b**) MoS_2 and (**c**) CoTe.

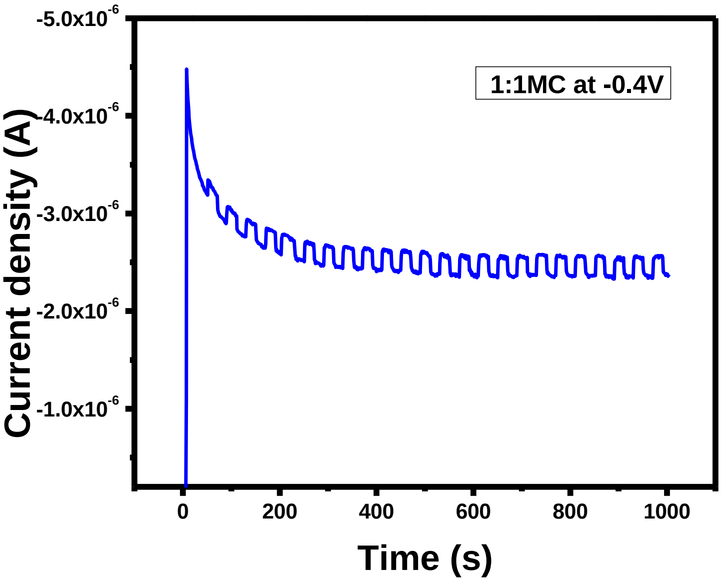

Figure S4. Chronoamperometric response of light on/off for 1:1 MC.
